# Formation of sweet potato starch nanoparticles by ultrasonic—assisted nanoprecipitation: Effect of cold plasma treatment

**DOI:** 10.3389/fbioe.2022.986033

**Published:** 2022-09-16

**Authors:** Jian Wang, Yu-Die Yu, Zhi-Guo Zhang, Wei-Cheng Wu, Pei-Long Sun, Ming Cai, Kai Yang

**Affiliations:** ^1^ College of Food Science and Technology, Zhejiang University of Technology, Hangzhou, Zhejiang, China; ^2^ Food Science Institute, Zhejiang Academy of Agricultural Sciences, Hangzhou, Zhejiang, China

**Keywords:** sweet potato, starch nanoparticles, cold plasma, nanoprecipitation, ultra sonification

## Abstract

Starch nanoparticles (SNPs) were produced from sweet potato starches by ultrasonic treatment combined with rapid nanoprecipitation. The starch concentration, ultrasonic time, and the ratio of starch solution to ethanol were optimized through dynamic light scattering (DLS) technique to obtain SNPs with a Z-average size of 64.51 ± 0.15 nm, poly dispersity index (PDI) of 0.23 ± 0.01. However, after freeze drying, the SNPs showed varying degrees of aggregation depending on the particle size of SNPs before freeze-drying. The smaller the particle size, the more serious the aggregation. Therefore, we tried to treat SNPs with dielectric barrier discharge cold plasma before freeze drying. Properties including morphological features, crystalline structure and apparent viscosity of various starches were measured by field emission scanning electron microscopy (FE-SEM), X-ray diffraction (XRD), and rheometer, respectively. The results showed that, after cold plasma (CP) treatment, the aggregation of SNPs during freeze drying was significantly inhibited. Compared to the native sweet potato starch, SNPs showed a higher relative crystallinity and a lower apparent viscosity. After CP treatment, the relative crystallinity of CP SNPs was further higher, and the apparent viscosity was lower. This work provides new ideas for the preparation of SNPs and could promote the development of sweet potato SNPs in the field of active ingredient delivery.

## 1 Introduction

Sweet potato starch is one of the richest sourced edible starches (Lyu et al., 2021). For broaden the field of application of sweet potato starch, physical, chemical, enzymatic or combined methods have been used to treat the starch in order to improve its structural and functional properties. Among these, preparation of starch nanoparticles (SNPs) is currently an important direction of analysis in the deep processing of starch. SNPs have been prepared through chemical, physical or biological methods to reduce the particle size of the original starch from the micron scale to the nanoscale. Nanoparticles have multiple beneficial attributes such as small size, more surface area, quantum size, and macroscopic quantum tunneling effect which are absent in conventional starches. Therefore, it is desired to prepare the nano-scale starch particles to improve their physical and chemical properties (Wang and Zhang, 2021). The performance of nano-materials like SNPs is strongly dependent on their characteristics, such as particle size, morphology, dispersion, microstructure, and rheology as well as how these characteristics change under different processing conditions (Perez Herrera et al., 2017; Dong et al., 2021; Li et al., 2021a; Li et al., 2021b).

There are two approaches for the preparation of SNPs, “top-down” and “bottom-up” approaches. The “top-down” approach uses physical or chemical means (hydrolysis, mechanical grinding, etc.) to prepare SNPs by decreasing the size of the starch particles. For example, SNPs were isolated from native starch granules by ball milling combined with acid hydrolysis (Ye et al., 2017). The “bottom-up” approach is to prepare SNPs by assembling starch molecules as basic units (e.g., nanoprecipitation and reverse-phase microemulsion). Among these methods, nanoprecipitation, also known as anti-solvent precipitation, has increasingly gained interest due to its simplicity and scalable potential (Martinez Rivas et al., 2017). In this method, a polymer is first dissolved in a solvent (good solvent) to form a homogeneous polymer phase, and then this polymer solution is transferred to another solvent (non-solvent) or the non-solvent is added to the good solvent in which the polymer is dissolved to cause the polymer to precipitate and form nanoparticles. Recently, ultrasonic assisted nanoprecipitation has been proved as an effective approach for producing SNPs with specific desired properties (Dong et al., 2022). However, after drying, the aggregation of SNPs would always happen, triggered by reducing surface energy to reach a steady state (Wang and Zhang, 2021).

In recent years, cold plasma (CP) treatment has been proved as an effective surface modification technique (cheap, eco-friendly, and non-thermal food processing technique) (Kopuk et al., 2022). CP consists of ultraviolet (UV) photons, ions, free electrons, and reactive species such as reactive nitrogen species (RNS) and reactive oxygen species (ROS), which is created by supplying different forms of energy like radio, electric fields, magnetic fields, and microwave frequencies (Wang et al., 2021; Laroque et al., 2022). Apart from general microbial and enzyme inactivation purposes, CP can effectively modify the food macromolecules through their interactions with reactive plasma species (Kopuk et al., 2022). For starch, CP treatment results in various surface modifications of starches by oxidation, etching, crosslinking, and depolymerization (Wongsagonsup et al., 2014; Gao et al., 2019; Sudheesh et al., 2019). However, there are few reports available on the modification of SNPs surface function by CP, the structural and functional changes of SNPs treated by CP were confirmed (Shen et al., 2022).

In the current study, we prepared sweet potato SNPs by ultrasonic-assisted dissolution of starch and subsequent rapid nanoprecipitation. SNPs were further treated with CP before freeze drying. We supposed that, SNPs will be electric charged by CP treatment, and the free radicals created by CP can cause skeletal breakage of SNPs, producing smaller fragments. The effect of CP on the preparation of SNPs was evaluated by the characterization of CP treated SNPs like multiscale structure, physicochemical, and rheological properties. This work could offer a novel idea for optimizing the preparation process of SNPs to inhibit the aggregation of SNPs during the drying process.

## 2 Materials and methods

### 2.1 Materials

Sweet potato starch was isolated from sweet potatoes obtained from Zhejiang Academy of Agricultural Sciences according to the method of Gani (Gani et al., 2012). The moisture content of obtained sweet potato starch was determined as 9.89% referring to GB5009.3-2016. Phosphate buffered saline (PBS) buffer (pH 7.4) used was from Adamas-beta (Adamas, Shanghai, China). Ethanol, sodium hydroxide (NaOH), and other chemical reagents (Shanghai Lingfeng Chemical Reagent Co., Ltd., Shanghai, China) were of analytical grade. All aqueous solutions were prepared in distilled water.

### 2.2 Preparation of starch nanoparticles

Sweet potato SNPs were prepared by ultrasonic-assisted dissolution of starch and subsequent rapid nanoprecipitation according to the method developed by (Ahmad et al., 2020; Dong et al., 2022) with some modifications. Briefly, the native sweet potato starch was dispersed in distilled water (2%, 5%, 8%, w/v) and gelatinized at 85°C for 30 min with stirring on a vortex mixer. The gelatinized starch paste was then sonicated for 5–25 min using a 20 kHz probe sonicator (JY98-IIIDN, Ningbo Scientz Biotechnology Co., Ltd., Ningbo, China) equipped with an ultrasonic horn and a tapered tip of 6 mm. The power output of 600 W and 3/5 s on/off pulses were applied to minimize heat generation. Nanoprecipitation was carried out by pouring sonicated starch paste solution rapidly into the ethanol (95%, v/v) under constant stirring. The volume ratio of starch solution to ethanol was 1:1, 1:2 and 1:5. After thorough mixing, an aliquot of the colloidal suspension obtained was diluted with distilled water for the analysis of the particle size and polydispersity index (PDI) by dynamic light scattering (DLS, see Section 2.5).

### 2.3 Cold plasma treatment

The cold plasma (CP) treatment was applied according to (Shen et al., 2022) with some modifications. Briefly, CP was generated by the dielectric barrier discharge (DBD) plasma apparatus (CTP-2000K, Nanjing Suman Electronics Co., Ltd., Nanjing, China), which was composed of a reaction cell (DBD-50), a voltage regulator, and a high frequency AC power (50 kV, 10 kHz). The SNPs were centrifugally separated and re-dispersed in PBS buffer to prepare SNP suspensions with a concentration of 5%. The prepared SNP suspensions were uniformly placed on the under-quartz medium (thickness: 3 mm) and put in a DBD reactor at room temperature. The plasma treatment conditions were as follows: the gas source was air, the distance of the two parallel electrodes was 1.3 cm, the input voltage was 90 ± 1 V, and the discharge was 1 A. The SNPs were treated for 90 s and named as CP SNPs.

### 2.4 Freeze-drying

The SNPs and CP SNPs were centrifugally separated and re-dispersed in PBS buffer to prepare SNP suspensions for freezing at −20°C overnight in a freezer. The frozen samples were then freeze-dried (Scientz-12N, Ningbo Scientz Biotechnology Co., Ltd., Ningbo, China) at −56°C for 2 days.

### 2.5 Particle size distribution

Particle size distributions of SNPs and CP SNPs were assessed by a dynamic light scattering instrument (Zetasizer NanoZS90, Malvern Instruments, Worcestershire, United Kingdom). The SNPs solution (0.01%, w/v) was prepared with distilled water and dispersed for 30 min with an ultrasonic cleaner (SB-300DTY, Ningbo Scientz Biotechnology Co. Ltd., Ningbo, China) for complete dispersion of particles. The pH of the suspension was adjusted to pH 7. The refractive index of water and starch were 1.33 and 1.53, respectively. The Z-average diameter and PDI were calculated by the Zetasizer software from three separate samples with five readings per sample.

### 2.6 Micromorphological features

The micromorphology of native starch, SNPs, and CP SNPs were observed using a field emission scanning electron microscopy (FE-SEM) (FESEM SU8220, Hitachi Ltd., Japan) at the accelerating voltage of 15.0 kV. Samples were sticked on a double-sided adhesive tape with the other side on a circular aluminum platform and coated with a thin layer of gold-palladium alloy.

### 2.7 X-ray diffraction

The crystalline characteristics of native starch, SNPs, and CP SNPs were determined using an X-ray diffractometer (Empyrean, PANalytical, Inc., Netherlands) at 45 kV and 40 mA Cu-Kα (λ = 0.1541 nm) radiations. The XRD measurements were scanned from 4° to 30° (2θ) with a scanning speed of 2°/min. The XRD diffractograms were collected using the MDI-Jade 6.0 software and the relative crystallinity (%) were calculated by the ratio between the areas of crystallin region and total area of the diffractogram.

### 2.8 Measurements of static rheological properties

SNPs, CP SNPs suspensions of 8% (w/v) and native starch paste solution of 2% (w/v) were prepared to satisfy the lowest viscosity that can be detected by the rheometer and then equilibrated at 25°C for 30 min before each test. The rheological tests were measured using a stress-controlled rheometer (Physica MCR300, Anton Paar GmbH, Stuttgard, Germany) with a cone and plate geometry (cone angle, 2°; diameter, 40 mm; truncation gap, 53 μm). The continuous shear tests were performed at 25°C to measure the apparent viscosity. The shear rate range was from 0.1 to 100 s^−1^ in an upward sweep followed immediately by a downward sweep from 100 to 0.1 s^−1^. Three sweep cycles were conducted consecutively in order to understand the relationship between the apparent viscosity and the shear rate of the SNP suspensions as well as their thixotropic behaviors.

### 2.9 Re-dispersion stability

Dried native starch, SNPs and CP SNPs were redispersed in distilled water and stirred for 1 h, then the dispersions were diluted to 5% (w/v) with distilled water in tubes, and the photographs of the tubes were taken in 0 and 30 min.

### 2.10 Statistical analysis

All measurements were measured at least in duplicate. Means and standard deviations were calculated and differences between means were determined with Fishers Least Significant Difference (LSD) test at *p* < 0.05 significance level (Statgraphics Centurion XV).

## 3 Results and discussions

### 3.1 Effects of preparation parameters on the particle size of starch nanoparticles

#### 3.1.1 Effect of starch concentration

**FIGURE 1:**
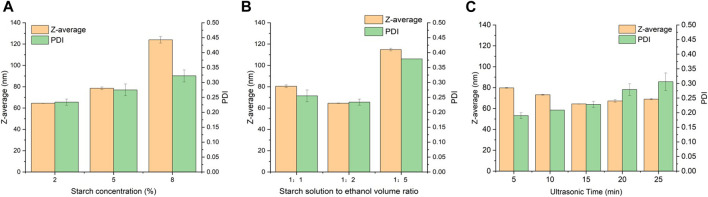
Effects of preparation parameters on the particle size of SNPs. **(A)** Starch concentration. **(B)** Starch solution to ethanol volume ratio. **(C)** Ultrasonic time.

Figure 1A shows the particle size and PDI of SNPs fabricated from different starch concentrations. With decrease in starch concentration from 8% to 2%, the particle size of SNPs decreased from 124.00 ± 11.74 nm to 64.51 ± 0.51 nm, and the PDI decreased from 0.32 ± 0.02 to 0.23 ± 0.01, indicating that the produced SNPs were smaller and more uniform. The ultrasound exerts an obvious impact on the structure of starch granules by the collapse of cavitation bubbles, disrupting starch granules, and the water diffusion breaks of the crystal structure (Zhu et al., 2012). Under the same sonication condition, the low concentration starch solution will be subjected to more forces and decomposed more completely, and it is easier to self-assemble into nanoparticles with smaller particle size. On the other hand, in the process of nanoprecipitation after ultrasonication, the higher concentration of starch solution corresponds to more short-chain starch molecules per unit volume, and the more starch molecular chains diffuse into ethanol during alcohol deposition, which are more likely to polymerize and entangle into nanoparticles with larger particle size. In addition, an increase in the concentration of the starch solution leads to a decrease in water holding capacity and an increase in viscosity and gelling, which further hinder the precipitation of the starch solution by ethanol. Similar result was found in other work (Hebeish et al., 2013), as the initial starch concentration increased from 2.5% to 10%, the particle size of the SNPs increased from 132 to 396 nm, and PDI increased from 0.401 to 1.000. It was further found by TEM images that the SNPs prepared with starch concentrations of 2.5% and 5.0% had a clear spherical shape and did not aggregate, while the groups with starch concentrations of 7.5% and 10.0% had a strong tendency to aggregate, and these highly aggregated particles caused an increase in PDI and elevated the instability of the solution. Lower starch concentrations (2.5, 5%) were able to increase the penetration of ethanol in the starch and improve the dispersion of the nano-starch by forming a dextrinized starch with relatively low viscosity.

#### 3.1.2. Effect of starch solution to ethanol volume ratio

As shown in Figure 1B, among the three starch solution to ethanol volume ratios, the SNPs with optimal particle size of 64.51 ± 0.15 nm, PDI of 0.23 ± 0.01 can be obtained at a starch solution to ethanol volume ratio of 1:2. Ethanol has a poor solubility for starch, so when starch solution is added dropwise to ethanol, a supersaturated solution of starch is formed, and the short-chain starch in the solution has a strong tendency to nucleate, and it is very easy to aggregate to form a double helix structure and then form starch granules. When the starch solution to ethanol volume ratio is low, little ethanol cannot create an environment of poor solvent, resulting in few nucleation sites and a slower growth trend of larger nanoparticles. Increasing the apportion of ethanol increases the diffusion distance during starch nucleation growth and limits the increase in nanoparticle size. Similar results were reported in other researches. (Wu et al., 2016) found that the average particle size of SNPs decreased from 213.1 ± 7.3 to 208.1 ± 1.0 nm, when the good solvent to poor solvent volume ratio changed from 1:5 to 1:30. (Kakran et al., 2012) found that as the good solvent to poor solvent ratio changed from 1:10 to 1:20, the length and diameter of nanoparticles decreased sharply from 1860 to 490 nm to 930 and 340 nm, respectively. When the ethanol ratio is too high, it may again make the repulsion in the solution too strong, the starch particles collision frequency increases, and excessive aggregation of starch particles leads to an increase in particle size. (Qiu et al., 2016) found that the particle size of SNPs was increased from 60 ± 1 nm to 92 ± 4 nm when the ratio of solvent to poor solvent was increased from 1:4 to 1:5.

#### 3.1.3 Effect of ultrasonic treatment duration

As shown in Figure 1C, the particle size of SNPs decreased from 79.73 ± 0.23 nm to 64.35 ± 0.15 nm during the variation of the ultrasonic time from 5 to 15 min, showing a stable decreasing trend, which can be explained by the fact that with the increase of the ultrasonic time, a stronger force is generated, which in turn breaks down the starch paste into smaller short-chain starches, which in turn self-assemble to form smaller SNPs. (Boufi et al., 2018) also found that the ultrasound treatment gradually reduced the particle size of standard starch from 1,200 nm and stabilized it at about 40 nm. (Chang et al., 2017) found that the size distribution pattern of SNPs prepared from un-sonicated starch solution had three peaks, located at 70 nm, 400 nm and 3–6 μm, respectively, with the peaks in the range of 3–6 μm indicating the presence of large particles or aggregates. The average size of SNPs decreased from 221.6 to 95.0 nm after 30 min of sonication, indicating that the increase of ultrasonic time could result in smaller and more uniform SNPs. However, when the ultrasonic treatment exceeded 15 min, the tiny starch fragments regrouped into new particles or adhered to the surface of the starch particles, which in turn formed larger starch particles, and these large particles led to a non-uniform particle size distribution of SNPs and increased the PDI. This result is in relative agreement with the results of (Wang et al., 2020a) for the ultrasonic treatment of sweet potato starch, which both showed the same trend of increasing and then decreasing.

### 3.2 Freeze drying of sweet potato starch nanoparticles

As shown in Table 1, the particle size and PDI of freeze-dried SNPs were generally increased with significant differences (*p* < 0.05) compared to the SNPs before freeze-drying. It indicates that freeze-drying intensifies the aggregation, recrystallization and widens the inhomogeneity of distribution of SNPs. The common theories currently used to explain this aggregation phenomenon include capillary pressure theory, chemical bond theory, and crystal bridge theory (Wang et al., 2005), the most accepted of which is the hydrogen bonding theory, which states that the presence of a large number of hydroxyl groups on the surface of starch nanoparticles makes them prone to self-aggregate in solution to form microaggregates under hydrogen bonding and van der Waals forces (Kim et al., 2015). Similar result was reported in other research (Dong et al., 2022), after freeze-drying, the SNPs of regular corn origin and fava bean origin increased from 166.9 to 232.5 nm, 107.6–152.5 nm, and the PDI increased from 0.28 to 0.29, 0.26 to 0.42, respectively.

**TABLE 1:** Mean particle size and PDI of SNPs obtained by different preparation parameters before and after freeze-drying.

Operation parameters			Before drying		After drying	
Ultrasonic time/min	Starch concentration /%	Starch solution to ethanol volume ratio	Mean size /nm	PDI	Mean size /nm	PDI
5			79.73 ± 0.23^cd^	0.19 ± 0.01^a^	254.6 ± 3.4^c^	0.51 ± 0.02^b^
10			73.16 ± 0.42^bc^	0.21 ± 0.00^ab^	290.2 ± 5.7^d^	0.34 ± 0.01^a^
15	2		64.35 ± 0.15^a^	0.23 ± 0.01^ab^	353.2 ± 8.6^e^	0.30 ± 0.08^a^
20			67.21 ± 1.27^ab^	0.28 ± 0.02^cd^	268.0 ± 8.8^c^	0.50 ± 0.03^b^
25		1:2	68.87 ± 0.62^ab^	0.31 ± 0.03^d^	261.3 ± 2.9^c^	0.54 ± 0.02^b^
15	5		78.54 ± 1.25^cd^	0.28 ± 0.02^bc^	194.4 ± 2.3^a^	0.67 ± 0.06^b^
	8		124.0 ± 3.05^e^	0.32 ± 0.02^e^	308.0 ± 13.8^d^	0.92 ± 0.04^e^
	2	1:1	80.41 ± 1.30^d^	0.26 ± 0.07^bc^	266.3 ± 6.6^c^	0.46 ± 0.04^b^
		1:5	114.8 ± 1.15^f^	0.38 ± 0.00^e^	233.5 ± 2.5^b^	0.33 ± 0.11^a^

Values presented as mean ± SD, indicate the replicates of three experiments.

Values with different letters (a, b, c, d, e and f) are significantly different (*p* < 0.05).

**FIGURE 2:**
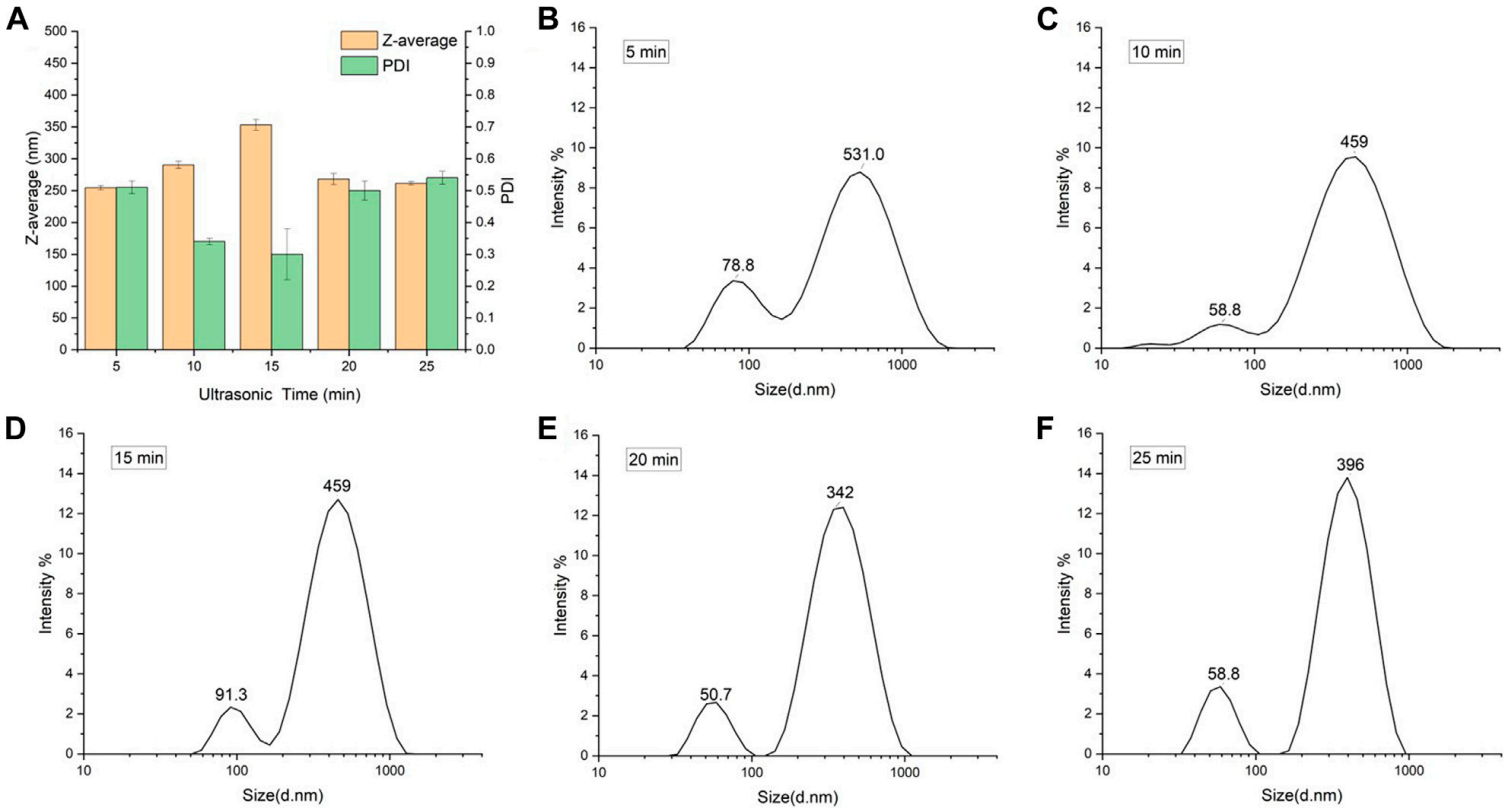
Particle size of freeze-dried SNPs prepared by different ultrasonic time. **(A)** Z-average and PDI. Size distribution of SNPs prepared by ultra-sonification for 5 min **(B)**, 10 min **(C)**, 15 min **(D)**, 20 min **(E)**, and 25 min **(F)**.

Observing from the variable of ultrasonic time (Table 1; Figure 2), it can be found that the increase in particle size and PDI after drying is related to the particle size of SNPs before drying, the smaller the particle size before freeze-drying, the larger the particle size and the smaller the PDI of the samples after freeze-drying. The smallest particle size of SNPs was 64.35 nm prepared by 15 min of ultrasonic time, and the particle size aggregated into the largest 353.20 nm after freeze-drying, while PDI was 0.30, it was the smallest among the PDI of all the freeze-dried SNPs, indicating that the smaller SNPs were more uniformly aggregated. The particle size of SNPs prepared by sonication for 5 min was the largest at 79.73 nm and aggregated into the smallest at 254.60 nm after freeze-drying. The same phenomenon was observed in other research (Sadeghi et al., 2017), when preparing SNPs using ethanol as a poor solvent. For investigating the effect of starch concentration, the ultrasonic time was kept same as 15 min, it was found that the PDI value of freeze-dried SNPs increased with increasing starch concentration from 2 to 8%, the span of particle distribution increased, and more insoluble large particles of starch appeared to collect at the bottom.

**FIGURE 3:**
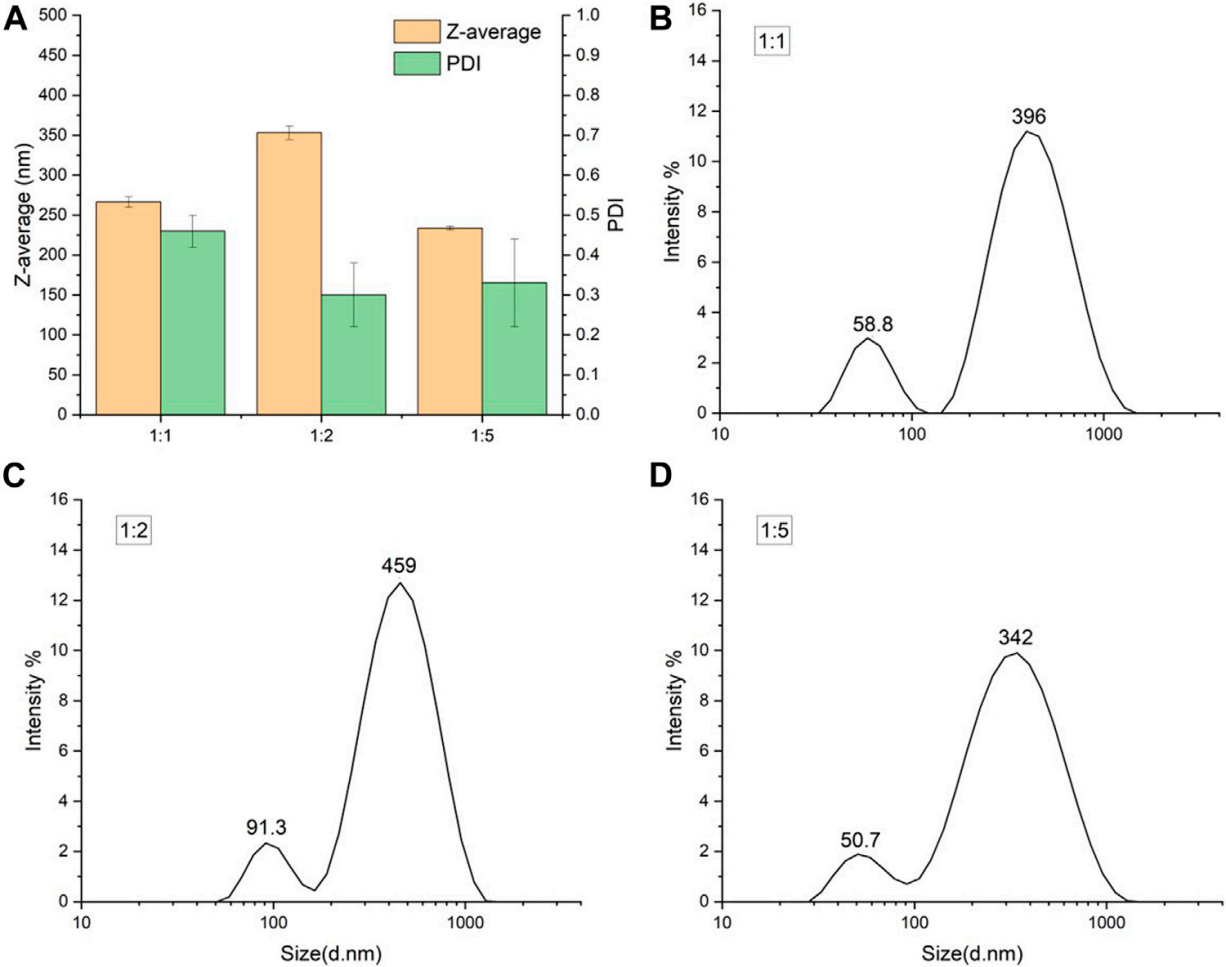
Particle size of freeze-dried SNPs prepared by different starch solution to ethanol volume ratios. **(A)** Z-average and PDI for three ratios. Size distribution of SNPs prepared at starch solution to ethanol volume ratio 1:1 **(B)**, 1:2 **(C)** and 1:5 **(D)**.

The starch solution to ethanol volume ratio also influenced the aggregation of SNPs (Table 1; Figure 3). The smaller size of SNPs also showed a stronger tendency to aggregate, with the freshly prepared SNPs having a particle size of 114.8 nm and PDI of 0.379 at a 1:5 ratio, and a particle size of 233.47 nm and PDI of 0.33 after freeze-drying and redispersion. (Dong et al., 2022) suggested that, the increase in the amount of ethanol caused the overgrowth of nanoparticles, because the supersaturation of starch molecules was too high for SNP formation, which in turn affects the redispersal of SNPs. When the starch solution to ethanol volume ratio is 1:1, freeze-dried SNPs show larger particle size and higher PDI, when the starch solution to ethanol volume ratio is higher (1:3, 1:5), the SNPs will show larger particle size and PDI. This phenomenon is contrary to our experiment, probably due to the fact that (Dong et al., 2022) prepared smaller SNPs when the starch solution to ethanol volume ratio was low, thus aggregating to produce larger particle size and narrower particle size distribution. In contrast, the particle size of the starch nanoparticles prepared in our experiment did not vary singularly with the starch solution to ethanol volume ratio; both too high and too low increased the particle size of the starch nanoparticles, so the similar trend was not observed.

### 3.3 Cold plasma treatment

**FIGURE 4:**
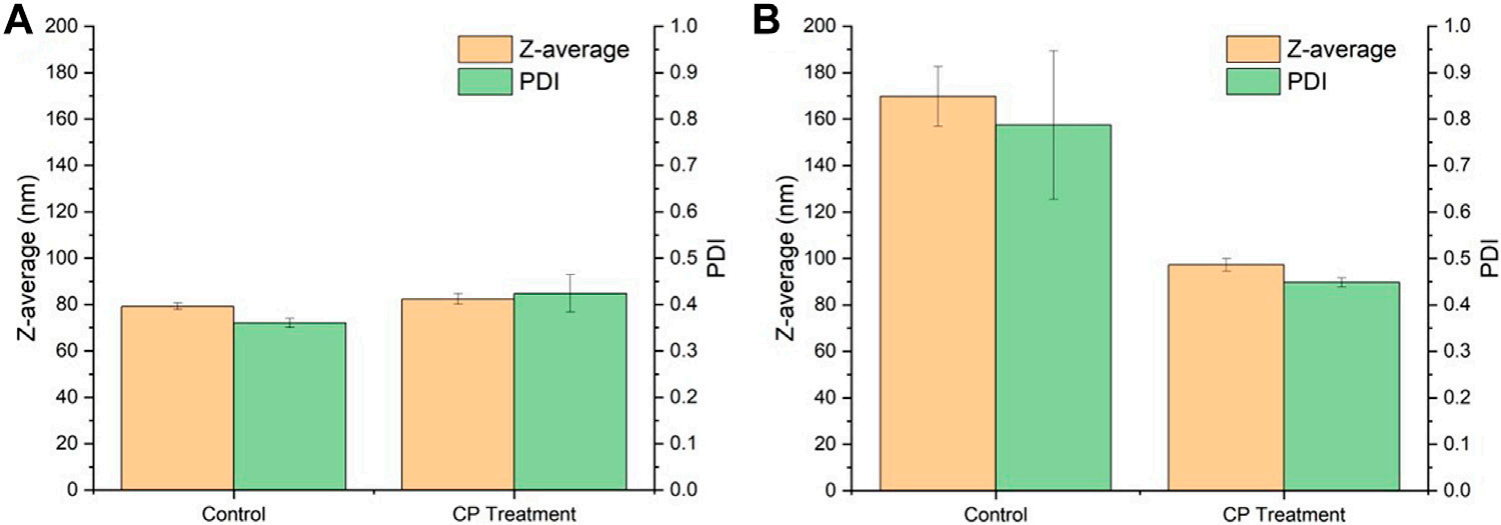
Effect of cold plasma treatment on the particle size of SNPs. **(A)** Before drying. **(B)** After drying.

Figure 4. shows the comparison of the particle size and PDI of SNPs before and after CP treatment. The particle size of the non-plasma treated group (control) increased from 79.19 to 169.80 nm and the PDI increased from 0.36 to 0.79, while the particle size of CP treated SNPs only changed from 82.41 to 97.30 nm and the PDI changed from 0.42 to 0.45, and no significant aggregation occurred. It indicated that there was no obvious aggregation phenomenon during freeze-drying. The SNPs before and after freeze-drying did not change much, and the CP treatment well inhibited the aggregation of SNPs during the freeze-drying process. (Chang et al., 2020) similarly found that the CP treatment changed the dispersion of SNPs and did not affect the particle size.

The depolymerization of starch by CP treatment has been widely reported (Gao et al., 2019; Shen et al., 2021), however, there are fewer studies on the effect of CP treatment on SNPs. The mechanism of plasma on starch is similar to the X-ray and gamma irradiation on it. Plasma treatment generates a series of highly active radicals, including superoxide radical (O^2−^), hydroxyl radical (OH·), peroxy radical (ROO·), alkoxy radical (RO·), free radical (NO·), hydrogen peroxide (H_2_O_2_), organic peroxide (ROOR'), ozone (O^3^), hypochlorite (HClO), singlet oxygen (^1^O_2_), aldehyde (HCOR) and peroxy nitrite (ONOOH). These free radicals cause skeletal breakage of polysaccharides, producing smaller fragments (Duan and Kasper, 2011).

### 3.4 Characterization of native starch, starch nanoparticles and cold plasma starch nanoparticles

#### 3.4.1 Field emission scanning electron microscopy of native starch, starch nanoparticles and cold plasma starch nanoparticles

**FIGURE 5:**
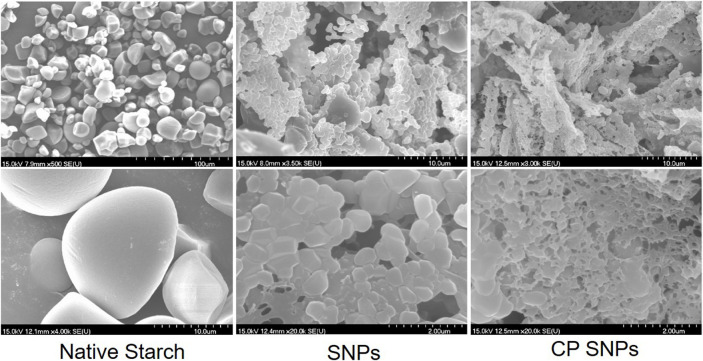
Field emission scanning electron micrographs (FE-SEM) of native starch, SNPs and CP SNPs.

The FE-SEM images of native starch, SNPs, and CP SNPs are shown in Figure 5. The surface and edges of the native starch particles are smooth, and the particles mainly exhibit round, oval, and polygonal shapes with particle sizes of 5–20 μm, which is consistent with other research (Kim et al., 2013; Wang et al., 2020b). Compared with the native starch, the adhesion between SNPs was more severe, and the adhered SNPs mainly showed a lamellar and spongy shape, which is similar to the shape of the SEM image obtained by (Dong et al., 2022). The adhesion phenomenon between SNPs may be due to the mutual attraction of hydrogen bonds between the particles, leading to tighter particle connections. The SEM images of SNPs showed that SNPs are mostly round and square in shape, the size of SNPs is in the range of 200–400 nm, which is basically in accordance with the particle size obtained by DLS. The freeze-drying process of SNPs is influenced by intermolecular and capillary forces, which force SNPs close to each other and bond by hydrogen bonding to reconstitute large granular crystals (Wang et al., 2005). Under the action of hydrogen bonds, these large particles will be automatically arranged, showing dense lamellar and spongy powders.

After CP treatment, the particle shape of CP SNPs completely disappears, interconnects more tightly and forms a tighter, more coiled fibrous network. (Charoenrein and Preechathammawong, 2012) also observed a spongy starch gel fibrous network in rice starch, and suggested that the formation of ice crystals during freezing and the aging of the starch were the main causes. Plasma treatment disrupts the functional groups on the surface of the SNPs and create new functional group. These new functional groups further affect the aggregation of the SNPs and promote the formation of gel webs.

#### 3.4.2 Crystalline structure

**FIGURE 6:**
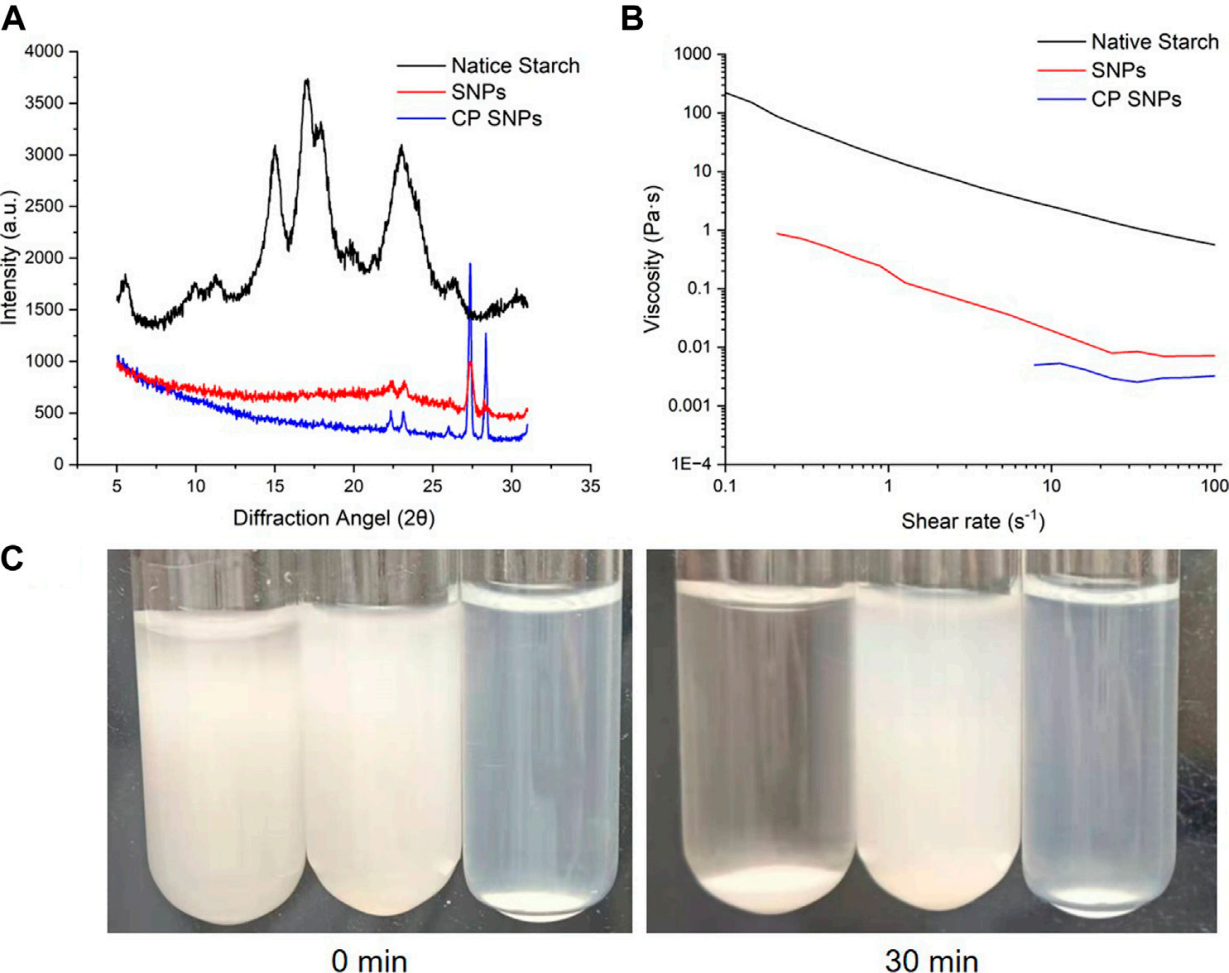
Properties of native starch, SNPs and CP SNPs. **(A)** X-ray diffraction patterns. **(B)** Apparent viscosity. **(C)** Photographs of re-dispersions of native starch (left), SNPs (middle) and CP SNPs (right) in water for 0 and 30 min.

The native starch, SNPs and CP SNPs were analyzed by X-ray diffraction analyser and the obtained diffraction patterns are shown in Figure 6A. The relative crystallinity of native starch, SNPs, and CP SNPs is 35.12%, 50.35% and 70.95%, respectively. It indicated that the crystalline region was destroyed during the preparation of SNPs and the crystallinity increased significantly (*p* < 0.05), while the plasma treatment further increased the crystallinity of starch (*p* < 0.05). In terms of characteristic peaks, strong diffraction peaks were observed at 5.6°, 17°, 18° and 23° for the naive starch (C-type starch), which is consistent with the reports about sweet potato starch (Kim et al., 2013; Wang et al., 2020b). After the formation of SNPs, all the diffraction peaks disappeared except the intensity of the diffraction peak at 23°, and new crystalline peaks were also observed on 22°, 27° and 28°, indicating that the crystalline shape of the starch changed significantly after formation of SNPs and new crystalline regions were generated. (Jhan et al., 2020; Jhan et al., 2021) similarly found disappearance of diffraction peaks and reduction in intensity when observing cereal SNPs. They attributed to the disruption of the ordered structure of branched starch during the ball milling preparation, resulting in the formation of more amorphous features in the starch granules. However, when ball milling of millet starch was performed to prepare SNPs, no crystallographic alteration was observed, and the diffraction spectra of SNPs were very similar to the native starch. The V-shaped starch crystal structure was mainly generated by the complex of straight-chain starch and ethanol, indicating that the cassava starch was ultrasonically alcohol deposited. (Ahmad et al., 2020) found that after preparation into SNPs, the peaks of the whole diffraction pattern all disappeared and became amorphous humps and the granules lost their crystallinity.

The crystallographic profiles of SNPs were almost unchanged after plasma treatment, and only the diffraction peaks at 22°, 23°, 27° and 28° became sharper, indicating that the plasma treatment further changed the crystal structure of SNPs. Except for sweet potato SNPs, no crystallographic changes were observed in rice starch (Thirumdas et al., 2017), corn starch (Sifuentes-Nieves et al., 2020), buckwheat, sorghum, wheat, and quinoa (Gao et al., 2019), when plasma was applied.

#### 3.4.3 Rheological and flow properties

Figure 6B represents the variation of viscosity with shear rate for native starch, SNPs and CP SNPs. The results showed that the viscosity decreased with shear rate increasing and all samples exhibited shear thinning behaviour. Starch solutions and SNPs solutions are mostly non Newtonian fluid and often exhibit shear thinning (Che et al., 2008), indicating the presence of an associated network structure between starch molecules. However, SNPs solutions at low concentrations may also exhibit shear thickening. (Kumari et al., 2022) attributed the shear thickening behaviour of SNPs solutions to the surface charge and repulsive double layer of the particles. When the shear rate was 30 s^−1^, the viscosity values containing native starch, SNPs and CP SNPs were 1.07, 0.008 and 0.003 Pa s, respectively. The viscosity of SNPs and CP SNPs was significantly lower than native starch. The same result was observed by (Kumari et al., 2022) in the preparation of SNPs, where the viscosity of the SNPs was consistently lower than that of the native starch throughout the flow curve, with a viscosity coefficient of 50.27 Pa·sn for 10% barley starch solution and only 0.0042 Pa·sn for 12% barley SNPs solution at a shear rate of 10 s^−1^, indicating the viscosity of the SNPs solution was significantly lower.

Plasma treatment can further reduce the viscosity of SNPs. (Shen et al., 2021) attributed this phenomenon to the degradation of starch chains during plasma treatment, which reduces the interaction of gel components. It was found that plasma treatment reduced the peak viscosity and final viscosity, and the longer the treatment time, the greater the decrease in viscosity. (Bie et al., 2016) found that the consistency factor of 8% starch solution decreased from 23.69 Pa·sn to 3.37 Pa·sn after 5 min of plasma treatment, while the shear thinning behaviour of plasma starch also showed less pronounced, and it was speculated that the plasma treatment may have caused oxidation and breakage of the starch particles, reducing the water holding capacity and increasing the solubility, which in turn changed the rheological behaviour. This low viscosity nature of SNPs and CP SNPs will expand the application of starch in industry, since low viscosity starch solutions do not affect the viscosity of the product much even when blended into liquid food products and are very suitable for the preparation of juices and beverages.

#### 3.4.4 Re-dispersion stability

Figure 6C shows the photos of native starch, SNPs, CP SNPs solutions with concentration of 5% at 0 min, 30 min, and found that the stability ranking was CP SNPs > SNPs > native starch. All of the native starch settled down after 30 min and the supernatant became completely clear water, indicating that the native starch was completely insoluble in water. The solubility of SNPs to water is better than the native starch, and it can be seen that it is basically still solution after 30 min, but there is also a mild sedimentation phenomenon. The bottom layer appears large particles of aggregates, the top layer solution becomes dilute and the solution colour becomes lighter. It is possible that by reducing the particle size of starch to the nanoscale, the bonds within and between hydrogen molecules are weakened and more hydroxyl groups are exposed, leading to more interactions with water molecules and thus increased solubility. (Jhan et al., 2021) found that at 90°C, the solubility of foxtail starch in water was 8.04% and sorghum starch was 9.08%, while the solubility of foxtail SNPs in water was 11.29% and 13.50% for sorghum nano starch, the water solubility of nanoparticles was generally greater. (Gonçalves et al., 2014) found that the solubility of araucaria angustifolia seeds starch in water increased from 3.27% to 16.90% after turning into starch granules. The water solubility of CP SNPs was the best, being a stable solution without delamination and precipitation. It may be due to the fact that CP treatment causes the SNPs to produce hydrophilic group carboxyl groups (Bie et al., 2016), which further enhances the stability of the solution. (Chang et al., 2020) found that CP treatment increased the absolute value of ζ-potential of the SNPs solution, and the SNPs solution exhibited slight precipitation when left for the same time at the same concentration, but the CP treated SNPs solution then remained stable.

## 4 Conclusion

In this research, sweet potato SNPs were prepared by ultrasonic-assisted dissolution of starch and subsequent rapid nanoprecipitation. After the optimization of preparation parameters, SNPs with mean particle size of 64.35 nm and PDI of 0.23 were obtained. However, the prepared SNPs would aggregate after drying. The results indicated that, SNPs with smaller size before drying would aggregate more seriously to become larger size after drying. For this, CP treatment was applied to SNPs before and after freeze drying. As a result, CP treatment would not change the particle size of SNPs before drying, but slowed down the aggregation and recrystallization of SNPs during the freeze drying. Among native starch, SNPs and CP SNPs, CP SNPs showed the lowest viscosity, the highest relative crystallinity and re-dispersion stability. Such a treatment strategy could offer an idea to inhibit the aggregation of SNPs, and the obtained CP SNPs could be used as nutraceutical or pharmaceutical carriers.

## Data Availability

The original contributions presented in the study are included in the article/Supplementary Material, further inquiries can be directed to the corresponding authors.
